# Genome-wide mapping of genomic DNA damage: methods and implications

**DOI:** 10.1007/s00018-021-03923-6

**Published:** 2021-08-31

**Authors:** Stefano Amente, Giovanni Scala, Barbara Majello, Somaiyeh Azmoun, Helen G. Tempest, Sanjay Premi, Marcus S. Cooke

**Affiliations:** 1 Department of Molecular Medicine and Medical Biotechnologies, University of Naples ‘Federico II’, Naples, Italy; 2 Department of Biology, University of Naples ‘Federico II’, Naples, Italy; 3 Department of Environmental Health Sciences, Florida International University, Miami, FL 33199 USA; 4 Department of Human and Molecular Genetics, Herbert Wertheim College of Medicine, Florida International University, Miami, FL 33199 USA; 5 Biomolecular Sciences Institute, Florida International University, Miami, FL 33199 USA; 6 Department of Tumor Biology, Moffitt Cancer Centre and Research Institute, Tampa, FL USA; 7 Oxidative Stress Group, Department of Cell Biology, Microbiology, and Molecular Biology, University of South Florida, 4202 East Fowler Avenue, ISA 6207, Tampa, 33620 USA

**Keywords:** Genomic instability, DNA damage, Adductomics, Next-generation sequencing, Mapping, DNA repair

## Abstract

Exposures from the external and internal environments lead to the modification of genomic DNA, which is implicated in the cause of numerous diseases, including cancer, cardiovascular, pulmonary and neurodegenerative diseases, together with ageing. However, the precise mechanism(s) linking the presence of damage, to impact upon cellular function and pathogenesis, is far from clear. Genomic location of specific forms of damage is likely to be highly informative in understanding this process, as the impact of downstream events (e.g. mutation, microsatellite instability, altered methylation and gene expression) on cellular function will be positional—events at key locations will have the greatest impact. However, until recently, methods for assessing DNA damage determined the totality of damage in the genomic location, with no positional information. The technique of “mapping DNA adductomics” describes the molecular approaches that map a variety of forms of DNA damage, to specific locations across the nuclear and mitochondrial genomes. We propose that integrated comparison of this information with other genome-wide data, such as mutational hotspots for specific genotoxins, tumour-specific mutation patterns and chromatin organisation and transcriptional activity in non-cancerous lesions (such as nevi), pre-cancerous conditions (such as polyps) and tumours, will improve our understanding of how environmental toxins lead to cancer. Adopting an analogous approach for non-cancer diseases, including the development of genome-wide assays for other cellular outcomes of DNA damage, will improve our understanding of the role of DNA damage in pathogenesis more generally.

## Introduction

**Fig. 1:**
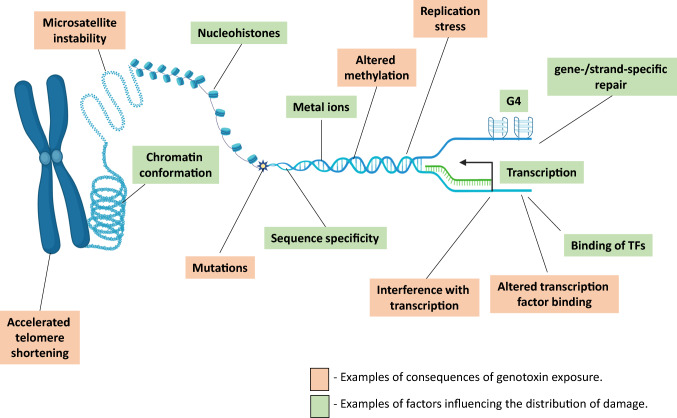
Examples of the factors influencing the distribution of damage and the potential consequences of genotoxin exposure. Many of the factors influencing the distribution of damage are inter-related; metal ions, sequence specificity, G-quadruplex secondary structures (G4), location within the nucleus, degree of chromatin condensation, presence of nucleohistones, gene-/strand-specific repair, transcription, chromatin conformation, DNA binding of TFs. Outcomes of genotoxin exposure include: mutation, microsatellite instability, altered methylation, altered transcription factor (TF) binding, telomere shortening, interference with transcription and replication stress

The presence of damage, in nuclear and/or mitochondrial DNA, has been linked with numerous diseases, including cancer, cardiovascular, pulmonary and neurodegenerative disease [1–7], and it is understood that exposure to the environment carries with it a risk of damage formation and with that risk of pathogenesis [8–10]. However, while elevated damage levels can be detected in disease states and damage has been shown to have downstream, functional effects that are clearly implicated in pathogenesis [(e.g. mutation [11, 12], microsatellite instability, altered methylation and gene expression [13], accelerated telomere shortening [14] (Fig. 1)], the mechanisms and sequence of events, linking DNA damage to disease in vivo is often unclear. Although we understand a great deal about the events between formation of DNA damage and the onset of disease, there remains something of a “black box”, which obscures our full understanding of the precise processes and hence limits our ability to intervene [15]. Herein, we aim to review an emerging field within the study of DNA damage, i.e. the mapping of DNA damage across the entire nuclear and mitochondrial genomes, with particular emphasis on the methods used and the factors which influence the distribution of that damage. We predict that this field will contribute towards addressing the issue of the black box and improve our understanding of the role of DNA damage in pathogenesis.

## Source and significance of damage to genomic DNA

Cells, tissues and organisms are under continual exposure from the internal [16] and external environments [17] (collectively termed the exposome [18]), which include stressors/agents that damage both nuclear and mitochondrial DNA [19]. Such exposures can lead to the formation of a wide variety of modifications, for example single- and double-strand breaks, alkali-labile sites [e.g. apurinic/apyrimidinic (AP) sites] and nucleobase modifications, such as 8-oxo-7,8-dihydroguanine (8-oxoGua), together with bulky modifications, such as cyclobutane pyrimidine dimers (CPD) and covalent adducts, such as DNA–DNA cross-links and DNA–protein cross-links. Such DNA damage can be referred to broadly as DNA lesions, as this term encompasses all modifications of DNA, from covalently modified adducts, to strand breaks and AP sites. However, to add a level of complication to the terminology, some modifications of DNA are “intentional”, i.e. arising from internal cellular processes, such as epigenetic modifications and yet may also be classified as damage when formed “unintentionally”, e.g. 8-oxoGua [20–22].

The amount and type of DNA damage present in human tissues reflect the nature, duration/size of exposures and processes such as activation, detoxification and repair [23]. While some forms of damage are specific for a particular stressor, such as CPD (which are generated from ultraviolet radiation), or 8,9-dihydro-8-(N7-guanyl)-9-hydroxyaflatoxin B1 (derived from dietary aflatoxin B1 exposure), they are invariably present in combination with other forms of damage, such as that derived from common pathways, such as oxidative stress [24], or endogenous cellular processes, such as spontaneous deamination, or methylation. Indeed, the term ‘damage’ might be regarded as inaccurate when referring to chemical alterations arising from certain endogenous processes, as these may be regarded as non-pathological, or even intentional events [22] and perhaps ‘modification’ is a more accurate term [20]. The consequence of these processes is a multiplicity of different types of alterations in DNA. In the case of oxidative stress, for example, there are over 24 major nucleobase products and the total number of lesions exceeds 100, when products of 2-deoxyribose and the phosphate backbone modifications are considered [25] and this does not include the lesions derived from secondary processes, such as lipid peroxidation [26].

This DNA damage, which may be mitigated by a network of DNA repair processes and other cellular defences, can lead to genomic instability and impact cellular function via a number of mechanisms, such as those reviewed by Evans and Cooke [14] and noted above. Through the disruption of cellular functions, nuclear and/or mitochondrial DNA damage plays a critical role in the pathogenesis of, arguably, all major human diseases, e.g. cancer, neurodegeneration and cardiovascular disease, plus ageing [25]. On this basis, the assessment of DNA damage is central to a wide variety of biomedical and related fields. These include: exposure biology and ecotoxicology [27, 28]; biomarkers (e.g. of oxidative stress) [29]; toxicity screening and testing; together with understanding pathogenesis (e.g. [30]). Furthermore, as human beings are exposed continually to a variety of potentially genotoxic chemicals across their life span and given that this exposure can be highly dynamic, there is a need for assessing accumulated exposures at all stages of life [31], not least because it is unknown which life stage is of particular importance for which disease.

## Methods for measuring DNA damage

### Assessment of nuclear vs. mitochondrial DNA damage

Most reports in the literature referring to the measurement of DNA damage relate to nuclear DNA or nuclear and mitochondrial DNA combined. This may be due to a perceived greater importance of damage to nuclear DNA or methodological reasons (most DNA extraction methods isolate both nuclear and mitochondrial DNA concurrently). Neither seem to be particularly adequate reasons. Mitochondrial DNA generally contains greater levels of damage than nuclear DNA due, in part, to its proximity to sources of ROS production, less protection from ‘sacrificial’ molecules (the smaller organelle contains fewer molecules than the larger nucleus) and a lack of higher-order levels of structure, which results in a vulnerability to damage formation from endogenous and exogenous sources [32, 33], despite protective pathways, such as DNA repair [34]. The proposed role of mitochondrial dysfunction [35] in diseases of numerous organ systems, such as the dermatological [36], immunological [37], cardiovascular [38] and in particular the neurological system [39, 40], is another compelling reason to study damage to mitochondrial DNA.

However, the challenges to the measurement of mitochondrial DNA damage include the requirement for additional steps to isolate mitochondria and the low levels of DNA obtained and hence damage, in isolated mitochondrial DNA, which is challenging to assess using the conventional methods described below. Methods for assessing mitochondrial DNA damage and repair have been considered elsewhere [15], which highlighted two recent descriptions of mapping DNA damage across the mitochondrial genome [41, 42] that are discussed further below.

### Assessment of global genomic DNA damage

Numerous methods exist for the quantification of DNA damage. As noted above, these methods can measure either nuclear or mitochondrial damage, depending upon the DNA extraction method used. Alternatively, through the extraction of both nuclear and mitochondrial DNA (which represents the majority DNA extraction methods), global genomic DNA damage is measured. Also, most methods perform a targeted analysis, measuring single, or a few, forms of DNA damage simultaneously [43]. Widely used methods include HPLC–MS/MS [44], the comet assay (single cell gel electrophoresis) [45, 46] and immunochemical methods [47], such as ELISA [48]. HPLC–MS/MS is widely regarded as the gold standard approach, providing target identification and absolute quantification. However, it can be time-consuming, with prescriptive sample workup steps, including isolation of cells, DNA extraction and acid or enzymatic hydrolysis, prior to analysis, which itself requires a relatively high level of expertise and expensive equipment. In contrast, due to its relative simplicity and lower equipment costs, the comet assay continues to gain increasing popularity as a means of quantifying DNA damage, repair and antioxidant capacity, in single cells from a wide variety of sources.

DNA adductomics is a paradigm-changing advance for the assessment of DNA damage, of which there are two variants. One form studies the totality of lesions in the genome, i.e., aims to comprehensively assess the totality and variety of damage, i.e. all the modifications of native nucleobases present in the genome (not just ‘adducts’, despite the assay’s title), using mass spectrometry. This approach is termed “cellular DNA adductomics” and is reviewed elsewhere [31, 49]. However, this approach is currently largely limited to monoadducts and cannot detect dimeric adducts (e.g. CPD) or modifications which form DNA intra- or inter-strand cross-links, or DNA–protein cross-links, although a method for DNA–DNA cross-links has been reported recently [50]. The other form of DNA adductomics maps the location of a single type of damage, e.g., CPD, or 8-oxoGua, across the genome (i.e. “mapping DNA adductomics”) and will be considered in further detail here.

### Mapping DNA damage across the genome

Despite the capability of the many techniques to quantify DNA damage and repair, they do not provide information on the genomic location of the damage and it is this information which is likely to give greater insight into the downstream, functional consequences of the damage. There exists several techniques which can locate damage and repair, within discrete genic and/or genomic regions. Initially, this approach was targeted towards individual genes, e.g., through the use of ligation-mediated PCR [51, 52] and immuno-coupled PCR [53, 54]. However, more recently, genome-wide mapping of damage has become possible (reviewed in [55]), most recently facilitated by the advent of next-generation sequencing. The earliest reports were limited to providing information at a chromosomal level only, with rather crude resolution [56], or offering little information in terms of gene-specific or intergenic regions [57]. In the last few years, there have been a growing number of reports in the literature describing methods for the genome-wide mapping of different types of DNA damage at high resolution. These methods include a series of approaches based upon combinations of excision repair enzymes and/or damaged DNA immunoprecipitation with microarray (DDIP-chip) or next-generation sequencing (DDIP-Seq). In the following section, for each type or class of DNA damage (summarised in Table 1), we describe and discuss a selection of DNA damage mapping methods, together with a summary of their most relevant biological findings.

**Table 1:** DNA damage products/modifications, to date, successfully mapped across the nuclear (and mitochondrial, where noted) genome and the method(s) used

DNA damage product	Source of DNA damage	Mapping method	References
CPD	Ultraviolet radiation	HS-damage-Seq	[58]
		DDIP-Seq (nuclear and mt genomes)	[42]
		Adduct-Seq	[59]
		XR-Seq	[60]
		CDP-Seq	[61]
		Excision-Seq	[62]
8-oxodG	Oxidative stress	OG-Seq	[65]
		AP-Seq	[66]
		OxiDIP-Seq	[67]
		Click-code-Seq	[68]
		enTRAP-Seq	[69]
M1dG	Oxidative stress	DDIP-Seq-based (nuclear and mt genomes)	[41]
Cisplatin-induced cross-links	Cisplatin	HS-damage-Seq	[70, 71]
		Cisplatin-Seq	[72]
		XR-Seq	[71]
BPDE-Gua	Benzo(a)pyrene	XR-Seq	[60]
AP sites	depurination; a by-product of DNA damage; failure of and intermediate in, DNA repair	AP-Seq	[66]
		snAP-Seq	[73]
		Nick-Seq	[74]
SSBs	By-product of DNA damage; failure of and intermediate in, DNA repair; topoisomerases activity	SSB-Seq	[75]
		SSiNGLe	[76]
		GLOE-Seq	[77]
DSBs	Topoisomerase activity; from closely located SSBs	BLESS	[78]
		BLISS	[79]
		DSB-Seq	[75]
		END-Seq	[80, 81]
		GUIDE-Seq	[83]
		Break-Seq	[82]
		LAM-HTGTS	[84]
Ura and ribonucleotides	Cytosine deamination; enzymatic misincorporation	Excision-Seq	[87]
		dU-Seq	[88]
		UPD-Seq	[90]
		U-DNA-Seq	[89]
		Endo-Seq/emRibo-Seq	[91]
		TrAEL-Seq	[158]
		Ad-Seq	[159]

## Mapping DNA adductomics

### UV-induced DNA damage, CPD and (6–4) photoproducts

CPD and (6–4) photoproducts [(6–4)PPs] are UV-specific forms of DNA damage and can be mapped at the genome-wide scale by means of the following six different techniques:

1. High-sensitivity damage sequencing (HS-damage-Seq) exploits the immunoprecipitation of CPD-containing DNA fragments, followed by a primer extension reaction with a biotinylated primer annealed to the 3' end of the CPD-containing DNA fragments. Damaged sites are then identified, during the primer extension reaction, as the sites where the DNA polymerase stalls. This approach has been used to perform genome-wide mapping of CPD and (6–4)PPs in UV-irradiated NHF1 human skin fibroblast cells [58].
2. DDIP-Seq has been used to map CPD across the nuclear and mitochondrial genomes of human HaCaT keratinocytes, with a resolution that is dependent upon the size (100–300 base pairs) of the sonicated DNA fragments [42]. DDIP-Seq uses a monoclonal antibody, specific to (Thy-Thy-containing CPD, T <  > T), to enrich for T <  > T-containing DNA fragments prior to NGS (Fig. 2). This study is of particular note, as it was the first to describe the distribution of T <  > T across the mitochondrial genome and noting it to be heterogeneous. Furthermore, the authors reported a time-dependent loss of T <  > T that was independent of mitochondrial turnover, implying that the T <  > T might be repaired, something also not previously described.

**Fig. 2:**
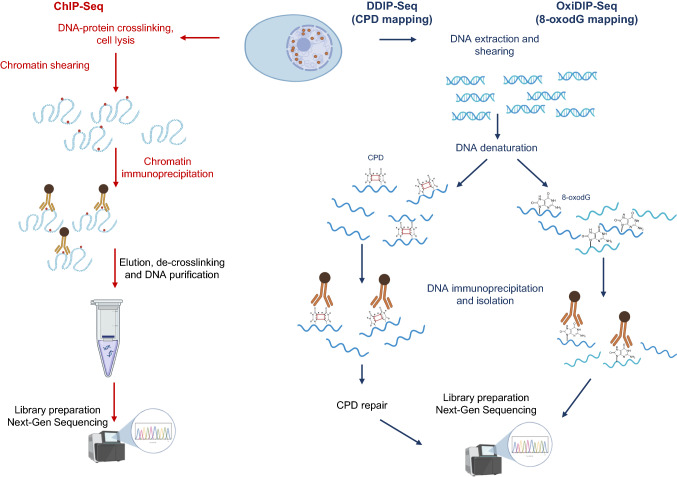
Overview of the methods for ChIP-Seq, DDIP-Seq and OxiDIP-Seq. Antibodies with the desired specificity are used to enrich chromatin (ChIP), or DNA (DDIP and OxiDIP) fragments containing the moiety of interest (e.g., proteins, T <  > T, or 8-oxodG, respectively), prior to NGS
3. Adduct-Seq has also been used to map CPDs with single-nucleotide resolution in primary human melanocytes. This method is based upon the ability of the T4 endonuclease V (T4endoV) enzyme to introduce a nick at the site of a CPD and on the sequential activity of: (i) photolyase, to remove the CPD; (ii) primer extension, to create a double-stranded end at the CPD site; and (iii) ligation with a biotinylated linker for purification of CPD-containing DNA fragments [59]. “Adduct-Seq” is highly versatile and can be used for high-resolution mapping of many adducts, single- and double-stranded breaks, AP sites and even 8-oxoGua, plus misincorporated nucleobases, such as uracil, simply by tagging the DNA nicks with a biotinylated linker. The nick can be introduced artificially, at the above-mentioned DNA adducts/nucleobase modifications, using enzymatic cleavage.
4. XR-Seq has also been used to map the occurrence of nucleotide excision repair (NER) events at CPD-containing sites in the genome, induced by UV treatment, in three types of skin fibroblast: NHF1, XP-C and CS-B. This approach combines the use of NER enzymes to excise CPD-containing DNA fragments, from the human genome, with DDIP performed using antibodies against CPD [60].
5. CPD-Seq and Excision-Seq have been employed to map UV-induced CPD in the yeast genome at a single-nucleotide resolution [61, 62]. These two methods differ from each other in the enzymes used to recognise the CPD site. In CPD-Seq, T4endoV and AP endonuclease 1 (APE1) are used to introduce a nick at the CPD sites, whereas in Excision-Seq, UVDE enzyme (ultraviolet endonuclease damage enzyme) is used to recognise and cleave the DNA immediately upstream of CPD. In both methods, the generation of a new 3′-end is ultimately used to ligate the adapter DNA that is needed for the subsequent sequencing step and the identification of CPD sites.

These methods have provided valuable information concerning the genome distribution, sequence context information and the repair process of CPD and (6–4)PPs, although there has been a discrepancy in some findings between the methods. For example, Excision-Seq analysis reported a uniform distribution of CPD and (6–4)PPs in the yeast genome, whereas the distribution was non-uniform, using CPD-Seq. All the methods highlighted the association between the presence of CPD, the sequence context and the repair process. Indeed, the transcription start site (TSS) regions (which are known to be nucleosome free) have been shown to contain fewer CPD, compared to other genomic regions [61, 62] and this has been associated with the presence of higher levels of transcription factor binding. Based upon these findings, it has been suggested that such binding to the TSS regions may have a protective role for such regions and be linked to the higher rate of repair activity which characterises these regions. Most recently, datasets from XR-Seq, Damage-Seq, Adduct-Seq, and CPD-Seq have been integrated to give a comprehensive assessment of UV-induced DNA damage and repair [63].

### 8-oxo-7,8-dihydro-2′-deoxyguanosine

8-oxo-7,8-dihydro-2′-deoxyguanosine (8-oxodG and its nucleobase equivalent, 8-oxoGua) is a major biomarker of oxidatively damaged DNA [64]. This modification has been mapped at a genome-wide scale in mouse, human and yeast during the last five years, with five different methods reported, based on the combination of next-generation sequencing technology with different affinity enrichment assays:

1. OG-Seq was used to map 8-oxodG in MEF cells [65]. In this method, 8-oxodG is selectively and chemically labelled with biotin, which is then used to enrich 8-oxodG-containing DNA fragments by means of a pull-down assay.
2. OGG1-AP-Seq is based on an in vitro enzymatic excision of 8-oxodG, from fragmented DNA genome, by using OGG1 enzyme followed by the biotin tagging of the resulting AP site (apurinic-apyrimidinic site) by using an aldehyde-reactive probe (ARP). Then, biotin-tagged DNA fragments are pulled down with streptavidin-coated magnetic beads [66].
3. OxiDIP-Seq is an ssDNA-immunoprecipitation-based enrichment assay based on an antibody raised against 8-oxodG [67] (Fig. 2).
4. Click-code-Seq exploits the specificity of DNA repair enzymes to substitute an 8-oxodG with a synthetic modified nucleotide (O-3′-propargyl-dGTP). Subsequently, a click DNA ligation reaction is used to label the modified nucleotide with a code sequence that is then used as a tag for affinity enrichment [68].
  AP-Seq and OxiDIP-Seq methods can achieve a resolution of ~ 300 bp, the latter mostly depending on the sonication method used for DNA shearing and have been used to map 8-oxodG in human (HepG2 for AP-Seq and MCF10A for OxiDIP-Seq) and mouse cells (MEFs for OxiDIP-Seq). The Click-code-Seq identified and mapped 8-oxodG at single-nucleotide resolution in the yeast (*Saccharomyces. cerevisiae*) genome.
5. Enzyme-mediated trapping and affinity precipitation of damaged DNA and sequencing (enTRAP-Seq) is the latest method developed for mapping 8-oxodG at the genome-wide level. This method was published by Zou’s group [69] and was used to map 8-oxodG across the mouse genome (i.e. in MEFs). enTRAP-Seq is based on an affinity enrichment of 8-oxodG-containing DNA fragments by means of an His-tagged OGG1 K249Q mutant which, lacking glycosylase activity, “entraps” itself in a stable complex with 8-oxodG. After trapping, the reaction mixture is purified with immobilised metal affinity chromatography (IMAC). This method can locate 8-oxodG in the mouse genome with a resolution of ~ 100–1000 bp, depending upon the efficiency of the dsDNA fragmentase enzyme used to fragment the DNA.

Overall, despite the differences existing between all the methodologies developed to map 8-oxodG across different genomes, a common and consistent observation, derived from the application of these methods, is that 8-oxodG is not randomly distributed and its presence is linked to chromosomal and chromatin structures (i.e. open regulatory regions are more prone to be oxidised) and to active transcription process.

### Malondialdehyde-dG

The 3-(2-deoxy-β-D-erythropentofuranosyl)pyrimido[1,2α]purin-10(3H)-one adduct, also known as M1dG, is a DNA modification formed by the reaction of electrophilic species, such as nucleobase propenals and malondialdehyde, with the DNA. M1dG is capable of inducing substitution and frameshift mutations and recently has been mapped across the nuclear and mitochondrial genomes by means of M1dG antibodies and a DDIP-Seq-based method [41]. Unlike the heterogeneous distribution of T <  > T in the mitochondrial genome, noted by Alhegaili et al. [42], Wauchope et al., reported that M1dG was equally distributed across the mitochondrial genome [41], suggesting that damage distributions might be lesion specific, perhaps due to the mechanisms of formation. Additionally, no time course was studied, so the persistence and/or removal of M1dG from the mitochondrial genome was not reported.

### Cisplatin-dG cross-links

Cisplatin is a drug used to treat various solid tumours (e.g. breast, lung, brain and bladder cancers) because of its capability to form inter- and intra-strand DNA cross-links which, in turn, can cause the stalling of the DNA replication machinery and consequential DSBs. Cisplatin-induced cross-links have been mapped in the human genome using two methods:

1. HS-damage-Seq [70, 71] is a DDIP method that uses an antibody against the cisplatin to perform enrichment.
2. Cisplatin-Seq [72], on the other hand, uses the HMGB1 domain A protein, for the enrichment step, which has a specific affinity for cisplatin-induced DNA damage. In both methods, the enrichment step is followed by a PCR-based amplification. During this amplification step, the cisplatin–DNA damage induces a site-specific stalling of the DNA polymerase and the site of stalling is used to mark the site of the cisplatin-induced damage. Moreover, the HS-damage-Seq method has been combined with the XR-Seq [71] to map NER events linked to the cisplatin damage repair.

Data from both HS-damage-Seq/XR-Seq and Cisplatin-Seq suggest that sequence context and chromatin folding may contribute to the formation of cross-links induced by cisplatin. Sequence context analysis, performed using data from both HS-damage-Seq/XR-Seq and Cisplatin-Seq, revealed that cisplatin-induced damage occurs preferentially at the G–G dinucleotide and TSS regions. Additionally, the analysis of the influence of chromatin folding on the cisplatin-induced damage has been performed, combining data from HS-damage-Seq/XR-Seq with nucleosome occupancy data from the ENCODE database. This provided evidence of a slight decrease in cisplatin-induced damage at the nucleosome centre. In contrast, Cisplatin-Seq-based data revealed that the cisplatin-induced damage colocalises with nucleosome signals, suggesting that there is a preference for cross-links to form on the nucleosome. These apparent contradictions may be explained by the fact that HS-damage-Seq couples damage formation data with damage-specific NER events from XR-Seq, whereas Cisplatin-Seq does not consider the presence of repair events; and the higher cisplatin damage levels observed in nucleosome regions with the latter method can be explained by a lack of repair processes, rather than a preference for damage formation in that region. Finally, the analysis of Cisplatin-Seq data combined with ChIP-Seq (see Fig. 2 for an overview of the approach) data shows that cisplatin damage tends to occur at the DNA binding regions of the RNAPII and CTCF proteins. Overall, these data support the proposal that chromatin folding, transcription and repair processes, and all contribute to the accumulation of cisplatin damage.

### Benzo[A]pyrene diol epoxide-dG

The diol epoxide, benzo[A]pyrene diol epoxide (BPDE), is a carcinogenic product of the cellular metabolism of benzo(a)pyrene and has been associated with lung cancer in smokers. BPDE forms adducts with deoxyguanosine, forming BPDE-dG, which is repaired via the NER pathway. The NER events associated with the repair of the BPDE-dG adducts have been mapped using XR-Seq [60]. Mapping data for BPDE-dG adducts provides evidence that such damage occur with a higher frequency at the CG dinucleotide sequence context, but it remains unclear whether this is due to preferential damage formation or decreased repair at these sites.

### Apurinic/apyrimidinic sites

Abasic or apurinic/apyrimidinic (AP) site, are locations in the DNA that have spontaneously, or after damage, lost a purine or pyrimidine nucleobase. In the genome, AP sites are also generated as intermediates of the BER process. Recently, the AP sites have been mapped, using three methods:

1. AP-Seq [66], already described for 8-oxodG genome mapping, was originally developed for genome mapping of AP sites. In this case, AP sites in genomic DNA fragments are directly biotin-tagged by using ARP, pulled down using streptavidin magnetic beads and prepared for high-throughput sequencing.
2. snAP-Seq [73], similar to the AP-Seq method for mapping 8-oxodG (described above), is a method that allows for the mapping of AP sites in the human genome by tagging of these latter with biotin and enriching biotin-tagged DNA fragments using a streptavidin-mediated pull-down assay. In contrast to AP-Seq, snAP-Seq has an alkaline cleavage step to increase its selectivity and achieve a single-nucleotide resolution in the mapping of AP sites.
3. Nick-Seq [74] is based on a strategy that increases both the sensitivity and specificity of AP site mapping. Briefly, genomic DNA is fragmented and the 3′-end of the DNA fragments are blocked with dideoxyNTPs by a terminal transferase. Then an AP endonuclease, endonuclease IV, is used to convert the AP sites contained within the 3′ tailed DNA fragments into single-strand breaks. Subsequently, using a complementary strategy, the 5′-ends of those DNA fragments are modified and subjected to a nick translation with α-thio-dNTPs to generate phosphorothioate oligonucleotides that are resistant to subsequent enzymatic hydrolysis of the bulk genomic DNA. This method allowed the identification and mapping of AP sites at single-nucleotide resolution in a bacterial genome (*Escherichia coli* and *Salmonella enterica* serovar Cerro 87).

All the studies performed with the above techniques report an association between the presence of AP sites and genomic regions linked to open chromatin conformation, transcription and replication processes. Notably, although snAP-Seq is, in principle, able to map AP sites at single-nucleotide resolution, it has not yet been able to identify the specific locations at which AP sites accumulate (at least in the human genome), if indeed they do. It appears that the reason for this is because AP site accumulation is a stochastic event and the consequential heterogeneity in the cell population makes it impossible to identify a well-defined hotspot of AP site accumulation.

### Single-strand breaks

SSBs represent the most abundant form of DNA damage and have been identified and mapped by a variety of methods:

1. SSB-Seq [75], which is a DDIP-based approach that uses an anti-digoxigenin antibody to enrich SSB-containing DNA fragments in which SSBs have been previously tagged with digoxigenin by a nick translation reaction.
2. Single-strand break mapping at nucleotide genome level (SSiNGLe) [76], in which the genomic DNA is first fragmented in situ with MNase and then the 3′-ends of the SSBs are tagged with a poly A tail using terminal transferase. The native DNA is then sequenced with two different platforms, Helicos Single Molecule Sequencing and Illumina HiSeq. SSiNGLe has been used to map SSBs in K562, N2a, HeLa and peripheral blood mononuclear cells.
3. Genome-wide ligation of 3′-OH ends, followed by sequencing (GLOE-Seq) [77] works by tagging the 3′-ends of the SSBs with biotin and enriching SSB-containing DNA fragments with a pull-down assay using streptavidin-conjugated beads. GLOE-Seq has been used to identify and map SSBs in *S. cerevisiae* and human (HCT116) cells.

Results from these methods suggested that SSBs are not randomly located and accumulate within promoter regions [75], regulatory elements [76] and in the leading strand during DNA replication, as a result of repair intermediates of ribonucleotide misincorporation [77].

### Double-strand breaks

DSBs can arise from replication and transcription stress, or from closely opposed SSBs accumulating within a specific DNA region; several techniques have been developed for their mapping across the genome:

1. Breaks labelling, enrichment on strepavidin and next-generation sequencing (BLESS) was the first method to map DSB [78]. BLESS is based on the labelling of DSBs with a biotinylated linker and their subsequent enrichment with streptavidin beads.
2. BLISS (breaks labelling in situ and sequencing) is a more versatile and sensitive method than the BLESS method and minimises the risk of introducing artificial DNA breaks during sample handling [79].
3. Other methods have also been developed to localise DSB in the human and yeast genomes [75, 80–84], as reported in Table 1. Many of these methods, which are similar to BLESS, are based upon the labelling of DSBs with a tag which is in turn utilised for the enrichment and sequencing of DNA fragments containing DSBs. These methods allow the identification of DSBs associated with topoisomerase activity [75, 80], off-target cleavage activities of CRISPR RNA-guided nucleases [83], transcription–replication stress [82] and RAG (Recombination-activating gene) protein-induced damage at the VDJ regions of the Ig genes [81].

Studies using DSB-mapping methods provide interesting information on the location of endogenous DSB events and also help to identify the biological processes that may be involved in their formation. DSBs have been mapped to regions sensitive to replication stress, such as aphidicolin-sensitive regions (ASRs) [78] and chromosome fragile sites, located within genes transcriptionally induced by hydroxyurea [82], thus identifying unscheduled clashes between replication and transcription machinery as cause of DSBs formation and genome instability. Finally, the transcription process itself has been shown to be a source of DSBs, at oncogenic superenhancers [85], or upon release of paused RNA polymerase II, at promoter regions [86].

### Uracil and ribonucleotides

The presence of uracil in genomic DNA can result from misincorporation events during DNA replication and from cytosine deamination and has been mapped by several techniques:

1. Excision-Seq, as described above [87], highlights a close correlation between the accumulation of uracil and replication timing, supporting the observation that uracil is incorporated during the replication process, while the dU-Seq found the accumulation of uracil at centromeres in the human genome.
2. dU-Seq [88] and U-DNA-Seq [89]. dU-Seq and U-DNA-Seq have been used to map uracil in the DNA of human cells (K562, WPMY-1, HEK293T for the former and HCT116 for the latter method), while UPD-Seq has been applied to the mapping of uracil in the bacterial genome.
3. UPD-Seq [90]: both dU-Seq and UDP-Seq methods are based upon the removal (using the UDG enzyme) and substitution (with a biotin-containing molecule) of the uracil. In contrast, U-DNA-Seq uses a uracil-DNA sensor (a mutant form of the human BER glycosylases UNG2) to identify uracil in the genome of human tumour cells treated with two drugs, raltitrexed and 5-formyl-deoxyuridine.

In addition to uracil, other ribonucleotides can be misincorporated into DNA, by several polymerases (e.g. DNA polymerases, PrimPol and RNA primase). Given their ability to generate DNA damage (strand breaks and cross-links, for instance), ribonucleotides represent a threat to genome integrity and to map them several genome-wide methods have been developed (Table 1). Among these, emRibo-Seq [91] is able to map the genome-wide distribution of these “embedded” ribonucleotides and uses the recombinant RNase H2 to cleave the embedded ribonucleotide, creating a break, which can be mapped subsequently. emRibo-Seq has been used successfully to determine that, in the *S. cerevisiae* genome, ribonucleotide incorporation is non-random and replication-associated. Endo-Seq has been adapted from emRibo-Seq, by replacing RNase H2 with specific nicking endonucleases (i.e. Nb.BtsI, RNase HII) and has been used for the genome-wide mapping of other non-canonical bases and to map endonucleases-generated ends in vitro [92]. Finally, it is also worth noting that methods such as emRibo-Seq and GLOE-Seq can be adapted to a variety of lesions, making them more versatile than other specialised methods, based on antibodies, or lesion-specific features. However, approaches that rely upon the availability of enzymes to detect a particular moiety, such as emRibo-Seq, are only as versatile as the substrate specificities of the available enzymes. Furthermore, some enzymes do not have narrow substrate specificities and recognise multiple moieties, e.g. formamidopyrimidine-DNA glycosylase [93], which may limit their usefulness.

Methodologies for the mapping of damage across the genome, such as those described above, have largely noted a differential distribution of damage across the entire genome, suggesting that certain regions of the genome are more vulnerable to damage and/or are more refractory to repair. This phenomenon has resulted in the suggestion that these key sites may be present within persistent DNA damage foci (PDDF), regions of unrepaired DNA, contained within protein assemblies, comprising elements of the DNA damage response [94]. In the sections to follow, we consider some of the factors that influence the distribution of damage and repair.

## Factors influencing the distribution of DNA damage

It is important to note that the mapping methodologies described above do not examine the three-dimensional (3D) spatial organisation of chromatin. The genome is not a two-dimensional linear structure, rather it is a dynamic and highly structured entity that contains different domains, formed by the association and interaction of DNA with various histone and non-histone proteins and protein modifications, to form chromatin [95]. The 3D organisation of chromosomes in the interphase nucleus is highly compartmentalised, with chromosomes forming chromosome territories (CTs) that are non-randomly organised in the nucleus [96]. This higher-order genome architecture has been shown to be cell-type specific and evolutionarily conserved [96]. The spatial organisation of the genome alongside the chromatin landscape, plays a critical role in a whole host of nuclear transactions including DNA replication, repair and recombination [95]. CTs also exhibit organisational plasticity, i.e., active gene domains are preferentially positioned where neighbouring CTs intermingle with each other and nuclear structures, within the inter-chromosomal spaces [97]. The spatial arrangement of CTs has been shown to influence the outcome and frequency of chromosome translocations following DNA damage and repair between two or more chromosomes, at a frequency higher than would be expected at random [97–99]. Thus, the spatial organisation of the genome likely plays a critical role in where DNA damage accumulates and structural reorganisation likely assists the DNA damage response by assisting cell cycle arrest, altering the transcription profile and allowing for conformational changes that allow greater accessibility of repair mechanisms to sites of DNA damage [100]. Some of the above findings have been established using DNA-FISH (fluorescence in situ hybridisation), but it is also important to note the existence of proximity ligation-based chromosome conformation capture (3C) techniques, such as Hi-C [101]. Such methods and the recent development of ligation-free approaches, such as genome architecture mapping, split-pool recognition of interactions by tag extension (SPRITE) and chromatin-interaction analysis via droplet-based and barcode-linked, sequencing (reviewed in [102]) not only help to discover new aspects of 3D genome topology, but also may offer the potential to bridge the gap between sequence context and spatial relationships within the nucleus.

The application of mapping technologies to study the origins of the DNA contained within PDDFs [94] is illustrative of how such methods may aid in our understanding of pathogenesis; for example, the accumulation of DNA lesions and subsequent loss of genome integrity is associated with ageing, many neurodegenerative disorders [103] and cancer. Additionally, changes in nuclear position of genes and CTs have been reported in a number of diseases including cancer [104] and ageing. However, further information is required to elucidate fully the factors which influence the distribution of DNA damage and hence the downstream consequences. Below, we have divided these factors into (1) formation and (2) repair of damage, largely for convenience/clarity, as some factors are clearly different, but some also show considerable overlap.

1. Factors influencing where damage is formed.
  The induction of damage by UVR depends upon the DNA sequence, local structure and chromatin environment/organisation [105, 106], together with protein interactions. For example, spatial repositioning of CTs has been observed in human lymphocytes following exposure to UVB [107]. More broadly, these will contribute to the expected, differential distribution of damage formation induced by many DNA damaging agents. It might be thought that ionising radiation is a notable exception to this, as the track of ionisation, as it passes through the cell, gives rise to discrete energy depositions, influenced by local oxygen status and this determines the distribution of damage [108], rather than more structural factors. The result is the characteristic clusters of lesions, signature to ionising radiation, which underlie the deleterious biological consequences of ionising radiation [108]. However, while there is some evidence to suggest that this is the case for β-form DNA, conformation and certain structural features of DNA can influence the distribution of SBs [109] and the presence of local hydroxyl radical (^•^OH) scavengers can limit [110], or influence, the final location of the damage. Finally, irrespective of whether the distribution of ionising radiation-induced damage is random, chromatin organisation can also have a major impact on the cellular response [111], including protection and repair, of DNA [112]. This particular subject is reviewed by Falk and Hausmann (2020), who also discuss “newly emerging super-resolution techniques”, but refer to optical super-resolution microscopy (nanoscopy) techniques, rather than DNA adductomics [113]. Another perspective on chromatin organisation, which has been explored in detail by Friz Thoma, relates to nucleosome organisation [114, 115]. The core histone proteins restrict DNA–protein interactions, which include transcription factors, DNA repair proteins and polymerases [116]. These authors also described the dynamic nature of nucleosomes, which modulates damage and allows rapid repair on the outer surface of the nucleosome, followed by the central regions of the nucleosomes. However, the influences of cell cycle, levels of DNA damage and the nature of the damaging agent are other factors which should be considered when considering the heterogeneity of damage. In our unpublished reports, we aimed to explore whether “dark” CPDs (i.e. formed after exposure to UV has been removed [59, 117, 118]) were more prevalent on the outer surface of nuclear DNA, because of their proximity to oxidised melanin in the cytoplasm. However, we noticed a homogeneous distribution of the CPDs throughout the nucleus, as determined by three-dimensional, confocal microscopy. We therefore proposed that the distribution of dark CPD depends, in part, on the dynamic properties of the nuclear DNA, not least due to its anchoring to the nuclear membrane [119–121]. However, this remains to be tested experimentally. Nuclear/nucleosome organisation is envisaged to impact the kinetics of repair in a similar manner.
  In the case of ROS-induced damage and H_2_O_2_-/O_2_^•−^-induced damage in particular, the availability and location of transition metal ions (e.g., copper and iron) and copper has a close association with DNA [122]. This will influence where the metal-catalysed Haber–Weiss reaction will lead to local production of the highly reactive and damaging, ^•^OH [123], with the copper-catalysed Haber–Weiss reaction causing more damage than iron (i.e., the Fenton reaction) [124, 125] and the reaction occurring at a faster rate if the metal ions are reduced [126]. Although the significance of ^•^OH has recently been called into question, in favour of the carbonate radical cation [21], given that the carbonate radical anion is formed from the Fenton reaction, the case for site-specific damage still applies. Furthermore, as it can migrate over long distances in duplex DNA, ultimately generating 8-oxoGua, it remains a factor influencing the distribution of damage [21]. Spatial repositioning of CTs in human lymphocytes following DNA damage induced by exposure to H_2_O_2_ has also been reported. Interestingly, when compared to UVB exposure, differences in both the chromosomes involved and spatial repositioning were observed [107]. This suggests that the type of DNA damage induced results in differences in mobility and/or decondensation of chromatin, chromatin regions affected and DDR.
  Local DNA sequence is also a factor in susceptibility to damage, such as G-quadruplex-forming sequences, in the case of oxidatively generated damage [21] (perhaps unsurprisingly as Gua is the most easily oxidised nucleobase) and the human telomeric repeat unit (5′TTAGGG/CCCTAA3′) which is nearly optimal for the induction of UV-induced CPD [127].
  Other genomic regions which confer sensitivity to damage formation include active transcription factor binding sites (TFBS), specifically those at which E26 transformation-specific transcription factors (ETS TFs) are bound to the DNA [128]. The binding of ETS TFs results in structural changes to the DNA, the nature of which is not clear (although clues have been suggested previously [129]) and this promotes the formation of CPD. This results in elevated levels of CPD, following irradiation and the presence of the unique signature of CPD hotspots, that are highly correlated with recurrent mutations in melanomas, despite (or perhaps because of?) high repair activity at these sites [128]. Further to this, a recent report, studying the rate of formation and repair of CPD within the TFBS of different TF families, noted an increased rate of formation of CPD associated with the tryptophan cluster family specifically [130]. However, for most TF families the increased mutation rate within the entire DNA region covered by the TF protein results from the persistence of lesions, rather than increased induction of damage [130]. Besides TFBS, gene promoters are particularly prone to accumulate oxidatively generated damage to DNA [13]. Indeed, although it is still unknown whether the sensitivity of promoters to oxidatively generated damage is dependent on an increase in the rate of formation, or decrease in repair, the accumulation of 8-oxodG at promoter regions has been linked to transcription and/or replication processes as well as to secondary DNA structures as R-Loops and G-quadruplex [13, 22]. Nevertheless, this illustrates additional factors which influence the distribution of damage and repair (see below).
2. Factors influencing where repair occurs.
  Bridging the factors influencing both DNA damage formation and repair are transcription and chromatin states. This is exemplified in a study of cisplatin adducts which demonstrated that the formation of damage is heterogeneous and that the effect of the overall accumulated damage driven not by damage formation, but by repair efficiency [71], at least in the case of cisplatin-induced adducts. Furthermore, the rate of repair on both the transcribed (TS) and non-transcribed strands (NTS) of expressed genes is positively correlated with gene expression. Indeed, it is suggested that transcription actually stimulates the repair of damage in the NTS as a result of transcription being associated with an open chromatin conformation, which provides the repair machinery greater access to the damaged DNA [71]. Large-scale CT spatial repositioning contingent upon double-strand break recognition and damage sensing has also been observed in human dermal fibroblasts following cisplatin exposure [100]. Critically, this study also demonstrated CT positioning reverted to pre-exposure locations following repair, suggesting an interplay between DNA damage sensing and CT relocalisation is an important aspect of DDR [100]. However, the adducts generated by cisplatin are not formed via normal endogenous processes and they are repaired by specific repair pathways, not BER which is responsible for the repair of the majority of non-bulky forms of DNA damage. This raises the question of how generalisable these results are to the effects of other lesions, even more so in the broader context of the diverse adductome of humans with a more complex exposome than laboratory mice. Nevertheless, it might be possible to assess the generalisability through comparisons with previous findings with other lesions and models. On the contrary, using exogenously cloned, nuclear and mitochondrial genes, Strand et al. demonstrated that the DNA damage distribution depends upon genomic sites, rather than repair efficiencies [131]. However, this might be attributed to the higher level of DNA damage in mitochondrial genome that persists longer owing to chronic ROS generation in the mitochondria [132].
  There is earlier evidence that cells prioritise the repair machinery to regions of specific need, to minimise disruption of function. For example, there are reports that repair is site specific, with preferential removal of DNA damage from transcriptionally active genes over inactive regions [133, 134] and TS-specific repair [134], processes which may be, in part, determined by nuclear organisation. Furthermore, the nature of the lesion influences whether, or not, there is preferential repair in transcriptionally active genes and in what stage of the cell cycle repair occurs [135], so the nature of the lesion itself is a factor. However, while repair of 8-oxoGua is more efficient in mitochondrial genes, compared to nuclear genes, in at least one study, no preference for transcribed genes over non-transcribed was noted, along with no strand preference [136]. Within genes, particular regions may be favoured, such as the 5′ portion of the DHFR gene [137], although the molecular basis for such differential repair across genes remains subject to speculation. This strand bias is now very well established, with transcription coupled-NER (TC-NER) being perhaps the most obvious example of a process leading to the biased removal of lesions [138], contributing to the heterogeneous distribution of damage and mutational, strand asymmetry in the cancer genome [139].
  In the case of UVR, chromatin structure and accessibility alter following exposure [140]. Furthermore, PDDF appears to occur more readily in repressive nuclear environments, such as in the perinucleolar domain, where they are frequently associated with Cajal bodies or heterochromatin [103] and in this instance it is possible to propose that lesion persistence occurs due to impeded access of the repair machinery, due to chromatin compaction and nuclear organisation. Indeed, as in the case of non-bulky lesions, such as 8-oxoGua, BER proteins are actively recruited to regions of open chromatin which, it has been suggested, leads to preferential repair of active chromosome regions [141]. Chromatin compaction leads to in a complete, but reversible, inhibition of the repair of 8-oxoguanine [141]. Collectively, these studies confirm that DNA repair is intimately associated with chromatin organisation and transcription and hence all three, together with “local DNA features” (such as frequently interacting regions and superenhancers [63]), play a role in the distribution of damage [142] and lead to super hotspots and super cold spots in the repair of CPD and (6–4)PP [63]. Importantly the above studies suggest that the findings of Yimit et al., [71] can be broadly generalised and apply to forms of DNA damage other than cisplatin-derived adducts.

## Features of a heterogeneous distribution of DNA damage: the example of ultraviolet radiation

On average, a normal cell sustains about a million alterations to its DNA in 24 h which, if not repaired, may lead to mutagenesis (amongst other outcomes) and ultimately diseases, including cancer. Out of the many pathologies, one of the increasingly well characterised is skin cancer, which is induced by exposure to environmental ultraviolet radiation. However, there have been few attempts at investigating and characterising the heterogeneity in the distribution of DNA damage in normal cells.

1. Damage and mutational tolerance.
  CPDs are the form of damage primarily responsible for UV-signature mutations present in sunlight-exposed skin and UV-induced melanoma [59]. Martincorena et al., describe the presence of a high levels of UV-signature mutations and by implication UV-induced DNA damage, in normal human skin [143]. Furthermore, targeted sequencing revealed a surprisingly high number of UV-induced mutations in 76 cancer-related genes in normal skin. This mutational landscape included ~ 73,904 base substitutions and 2248 small indels [143]. This study revealed that the mutations induced by an environmental carcinogen and hence the DNA damage can be well tolerated by normal cells until there is “a key mutational event” which drives a pathology, such as skin cancer. Damage, or mutational, tolerance must be widespread in the cell for in addition to the regions at which PDDFs assemble, other regions of the genome, such as at telomeres, also demonstrate almost negligible removal of CPD removal and cells containing persistent, high levels of telomeric CPDs nevertheless proliferate and chronic UV irradiation of cells does not accelerate telomere shortening [127]. These studies demonstrate that normal cells sustain DNA damage in heterogenous genomic regions without any pathological outcome, probably due to an insufficient burden of damage to initiate a pathological event, efficient repair, or that the location of the damage is in genomic regions that are not physiologically relevant for pathogenesis.
2. DNA regions hypersensitive to damage, refractive to repair.
  In 2019, Premi et al., discovered that the human genome has regions which are hypersensitive to the formation of CPDs [59]. This study describes sequence motifs preferentially damaged by UV, so-called CPD hyper-hotspots, located adjacent to ETS binding sites and 5’UTRs, or targets of the mTOR pathway. These CPD hyper-hotspots align well with the UV-signature mutation patterns in the same genomic regions, reported in melanoma [128]. Indeed, hotspots of CPD induction, or persistence, are more likely to yield mutations [144, 145] and about 80–90% of mutations in all human cancers can be correlated to regions of unrepaired DNA damage [146]. However, this does not mean that they are driver mutations, i.e. the rare selectively, positive mutations which increase a cell’s ability to proliferate more likely are passenger mutations, which occur at intrinsically mutable sites, under no positive selection [147].
  The presence of excessive levels of DNA damage, or densely, clustered damage, interferes with the recruitment of sensory proteins and thus DNA repair. Tobias et al., describe the spatiotemporal characteristics and repair kinetics, using radiation-induced double-strand breaks [148]. It was concluded that densely located DNA damage affects the recruitment of DNA repair proteins not only at the damage sites, but also at the sites distant from damaged DNA. This could be another factor contributing to the persistence, or distribution, of damage. Dense CPDs might affect the DNA directly through structural changes. Using synthetically designed oligonucleotides, previous studies have shown that the CPDs bend DNA by almost 30° which distorts DNA helix and destabilises the double-strand to single-strand equilibrium [149–151]. Such structural changes have been proposed to impart subtle effects on the biology of DNA [150, 151]. However, dense areas of CPDs at the hypersensitive genomic sites [59] might physically bend the DNA to such an extent that it de-regulates transcription, DNA repair and gene regulation, independently of the involvement of any proteins, although there is no experimental proof of this to date. Additionally, in response to condensed DNA damage, non-histone, epigenetic regulators such as DNA and histone methyltransferases and polycomb group proteins lead to dysregulation of the epigenome [152–154], as UV-induced adducts can induce acute loss of core and linker histones [155] leading to epigenetic reprograming. Coupled with other functional outcomes of the induction of DNA damage, such de-regulation might lead to hyper-aggressive disease phenotypes, including drug resistance and hyper-proliferative cancers.

## Conclusion

Mapping DNA adductomics is already well established as a promising tool to study a number of key aspects of DNA damage, such as: (A) the identification of genomic regions that are more (less) vulnerable to genotoxins that threaten DNA integrity. While the degree of chromatin condensation is a relatively well-known factor influencing the sensitivity to damage, local changes to DNA structure caused by protein binding is certainly worth further investigation; (B) to provide a standard tool, as part of human exposomic studies, to better characterise simple and complex exposures based upon the topography of damage. This, in particular, will require mapping DNA adductomics to be demonstrated to be applicable to human studies [e.g., suitable amounts of DNA can be obtained, relevance of surrogate tissues vs. target tissues (discussed in [15]) is address] and the assays are properly validated, in terms of establishing norms for the assays, such as assay variability and controls, intra- and inter-individual variability, age and sex differences, as has been performed for other biomarkers [156]; (C) the identification of potential environmental threats to public health; (D) the further elucidation of the “black box”, i.e., the nature, sequence and outcome of pathogenic, cellular events that occur between the formation of DNA damage and the onset of early- and late-stage disease. The goal is to elucidate the type and sequence of events that occur between damage formation and the onset of disease. This remains challenging, as even the mechanisms linking genomic alterations to transcriptional changes in cancer, which is part of this sequence, remain elusive [157]. This will require integration of multiple approaches, many of which will need to be ‘omics in nature, as co-analysis of mutational and gene expression profiles have shown [157]. Yet, additional levels of co-analysis will be required and DNA adductomics, both mapping (including the 3D spatial mapping) and cellular DNA adductomics (the totality of lesions), will both make a valuable contribution to this, particularly if the two approaches can be combined into an approach which maps the totality of lesions, across both the nuclear and mitochondrial genomes [15].

## Data Availability

Not applicable.
